# Effectiveness of perioperative low-dose esketamine infusion for postoperative pain management in pediatric urological surgery: a prospective clinical trial

**DOI:** 10.1186/s12871-024-02450-8

**Published:** 2024-02-15

**Authors:** Yanle Xie, Zenghui Liang, Shuhan Chen, Jing Liu, Huimin lv, Fei Xing, Yuanyuan Mao, Yanling Ren, Xin Wei, Zhongyu Wang, Na Xing, Jianjun Yang, Xiyao Gu, Jingjing Yuan

**Affiliations:** 1 Department of Anesthesiology, Pain and Perioperative Medicine, the First Affiliated Hospital of Zhengzhou University, No. 1 Jianshe East Road, Erqi District, Zhengzhou City, Henan Province China; 2 Henan Province International Joint Laboratory of Pain, Cognition and Emotion, Zhengzhou, Henan Province China; 3 Department of Anesthesiology, Renji Hospital, Shanghai Jiao Tong University School of Medicine, Shanghai, China; 4 Key Laboratory of Anesthesiology, Ministry of Education, Shanghai Jiao Tong University, Shanghai, China

**Keywords:** Postoperative pain, Pediatric urology, Esketamine

## Abstract

**Background:**

Postoperative pain is common in pediatric urological surgery. The study assess the impact of perioperative intravenous infusion of low-dose esketamine on postoperative pain in pediatric urological surgery.

**Methods:**

Pediatric patients (*n* = 80) undergoing urological surgery were randomized into four groups. Patients in the control group were administered an analgesic pump containing only hydromorphone at a dose of 0.1 mg/kg (Hydromorphone Group 1, H1) or 0.15 mg/kg (Hydromorphone Group 2, H2). Patients in the experimental group were injected intravenously with 0.3 mg/kg of esketamine (Esketamine group 1, ES1) or equal volume of saline (Esketamine Group 2, ES2) during anesthesia induction. Esketamine 1.0 mg/kg and hydromorphone 0.1 mg/kg were added to the analgesic pump. Face, Leg, Activity, Crying, and Comfort (FLACC) scale or the Numerical Rating Scale (NRS) and adverse effects were recorded at 2, 6, 24, and 48 h postoperatively. Additionally, total and effective PCA button presses were recorded.

**Results:**

In comparison to the H1 group, the pain scores were notably reduced at all postoperative time points in both the ES1 and H2 groups. The ES2 group exhibited lower pain scores only at 24 and 48 h postoperatively. When compared to the H2 group, there were no significant differences in pain scores at various postoperative time points in the ES2 group. However, the ES1 group demonstrated significantly lower pain scores at 6, 24 and 48 h postoperatively, and these scores were also significantly lower than those observed in the ES2 group. The total and effective number of PCA button presses in the ES1, ES2 and H2 group were lower than that in the H1 group (*P* < 0.001). The incidence of adverse effects within 48 h after surgery was 15% in ES1, 22% in ES2, 58% in H1, and 42% in H2, respectively (*P* = 0.021).

**Conclusions:**

The use of low-dose esketamine infusion in analgesia pump can effectively alleviates postoperative pain in pediatric urological patients, leading to a significant reduction in the number of analgesic pump button press. The combined approach of perioperative anesthesia induction and analgesia pump administration is recommended for optimal pain management in these patients.

**Trial registration:**

Chinese Clinical Trial Registry**-** ChiCTR2300073879 (24/07/2023).

## Introduction

Postoperative pain management in children is often insufficient, with a considerable percentage (25–63%) of patients experiencing moderate to severe pain on the day following urological surgery, which can persist for up to nine days [1]. Acute postoperative pain is recognized as a potential risk factor for chronic postoperative pain in children, and inadequate analgesia during the acute phase can contribute to the development of chronic pain [2]. Of 115 pediatric patients undergoing orthopedic, general and urological surgery, approximately 13% experienced chronic postoperative pain that affected their daily activities and sleep patterns [3].

A survey investigating the utiliazation of postoperative analgesics in pediatric urology revealed that opioids were frequently employed as part of the analgesic regimen [4]. Despite their effectiveness in pain management, opioids are associated with common adverse effects such as nausea, vomiting, constipation, and respiratory depression. Moreover, long-term use of high-dose opioids has been linked to hyperalgesia and immunosuppression [5, 6]. To address these concerns, the Pediatric Society of Anesthesiolog’s guidelines for perioperative children recommend the use of patient-controlled analgesia (PCA) as the preferred method over intramuscular and intermittent intravenous administration [7].

Postoperative pain management in pediatric urology lacks a definitive consensus, leading to the clinical recommendation of utilizing multimodal analgesia strategies [8]. Incorporating ketamine as an adjunct to multimodal analgesia has been shown to reduce the need for analgesics and improve postoperative pain scores [9]. Esketamine, an S-isomer of ketamine, non-competitively antagonizes N-methyl-D-aspartate (NMDA) receptors, and it offers superior in vivo clearance, anesthesia recovery quality, and incidence of adverse reactions compared to ketamine [10].

The main aim of this study was to evaluate the impact of perioperative intravenous infusion of low-dose esketamine on postoperative pain in pediatric urology. Additionally, the secondary objective was to explore the appropriate administration approach for esketamine.

## Materials and methods

### Study design

The study followed the CONSORT statement. This prospective, randomized, single-blind clinical trial was conducted at the First Affiliated Hospital of Zhengzhou University. The study protocol has been approved by the Ethics Committee of the First Affiliated Hospital of Zhengzhou University (2021-KY-1007-003) and registered in the China Clinical Trials Registry (ChiCTR2300073879). Informed consent was obtained from the parents of each participant.

### Patients

We recruited male participants aged 2 ~ 12 years who underwent elective urological procedures (urethroplasty, penile rectification, and laparoscopic pyeloplasty). The inclusion criteria were American Society of Anesthesiologists(ASA)grade I-II, body weight within ± 10% of the standard body weight, postoperative use of analgesic pumps, and informed consent of the patient’s parents. Exclusion criteria included severe organic lesions of vital organs such as heart, liver, and kidney; presence of psychiatric and neurological diseases that hindered cooperation; a history of chronic pain or analgesic medication before surgery; known allergy to anesthetic drugs or bronchial asthma; and history of acute upper respiratory tract infection within two weeks prior to surgery.

### Randomization and masking

Initially, the 80 patients were sequentially assigned numbers ranging from 1 to 80 based on the order of their visits. Subsequently, a 2-digit number was selected as the corresponding random number, starting from any row or column in the random number table. All generated random numbers were then arranged in ascending order. Patients with random number rankings from 1 to 20 were designated to the ES1 group, those with rankings from 21 to 40 were allocated to the ES2 group, individuals with rankings from 41 to 60 were assigned to the H1 group, and those from 61 to 80 were placed in the H2 group.

The study implementers administered the intervention based on the assigned groups; however, patients remained blinded to their respective groupings. In the case of the primary outcome, patients aged 8 years and above provided self-reported pain scores. For patients younger than 8 years, a third party, uninformed about the grouping, conducted postoperative pain assessments. Data collectors were responsible for gathering and documenting patient data. The interventions are outlined below:

Esketamine Group 1 (ES1) : Patients were administered esketamine at a dosage of 0.3 mg/kg during anesthesia induction. Subsequently, an analgesic pump was employed, incorporating 1.0 mg/kg of esketamine and 0.1 mg/kg of hydromorphone.

Esketamine Group 2 (ES2): Patients received an equal volume of saline during anesthesia induction. Subsequently, an analgesic pump was employed, incorporating 1.0 mg/kg of esketamine and 0.1 mg/kg of hydromorphone.

Hydromorphone Group 1 (H1): Patients were provided with an analgesic pump containing hydromorphone exclusively, administered at a dose of 0.1 mg/kg.

Hydromorphone Group 2 (H2): Patients received an analgesic pump exclusively containing hydromorphone at a dose of 0.15 mg/kg.

### Anesthesia methods

Anesthesia was induced with propofol 2 mg/kg, alfentanyl 20 ug/kg, and cisatracurium 0.2 mg/kg intravenouly. Following induction, a laryngeal mask was inserted for mechanical ventilation. Intraoperative monitoring included heart rate (HR), blood pressure (BP), pulse oximetry (SPO_2_), and end-tidal carbon dioxide (P_ET_CO_2_).

Anesthesia maintenance was achieved using 1.5-2% sevoflurane compounded with 0.1–0.25 µg/kg/min remifentanil. During the procedure, the drug dose was adjusted according to the patient’s vital signs, with fluctuations not exceeding ± 20% of the baseline value (baseline values were obtained before the start of anesthesia). Vasoactive drugs were given as needed. An volume-controlled ventilation mode was used during the procedure, with the tidal volume set at 6–8 ml/kg and the P_ET_CO_2_ was maintained between 35 and 40 mmHg.

Ketorolac 0.5 mg/kg (not to exceed 15 mg) was administered intravenously for postoperative analgesia 30 min before the end of the procedure. Once the patient resumed spontaneous breathing, the laryngeal mask was removed and the analgesic pump was connected.

The analgesic pump was prepared by diluting with saline to a total volume of 100 ml. The initial dose was set at 0 ml, the background infusion rate was set to 1 ml/ h, and the PCA dose was set to 2 ml per activation. A lockout time of 10 min was applied to ensure safe dosing. The analgesia was maintained through the PCA pump until 48 h after surgery.

### Data collection and outcomes

Postoperative pain in children was evaluated using either the Face, Leg, Activity, Crying, and Comfort (FLACC) scale or the Numerical Rating Scale (NRS). Children between aged 2 to 7 years were assessed using the FLACC scale, while children aged 8 to 12 years were evaluated using the NRS scale [11, 12]. If the FLACC or NRS score was ≥ 4, parents or healthcare providers assisted with PCA compression. If the FLACC or NRS score remained ≥ 4 after three or more PCA compressions, a remedy of 0.2 mg/kg ketorolac tromethamine was administered to enhance analgesia. The Ramsey Sedation Scale is one of the commonly used methods to assess the level of perioperative sedation in children [13–15]. Therefore, Ramsey Sedation Scale was used in this study to assess the level of postoperative sedation.

Baseline data of patients were recorded, including age, height, weight, ASA grade, intraoperative remifentanil consumption, intraoperative blood loss and fluid infusion. The main indicator of this study were FLACC or NRS pain scores at 2 h, 6 h, 24 h, and 48 h postoperatively. Secondary observations included Ramsay sedation scores and adverse effects in patients at 2 h, 6 h, 24 h, and 48 h after the operation. Additional data recorded included time to awakening and extubation, incidents of choking and agitation during removal of the laryngeal mask, time to first PCA compression after awakening, total and effective PCA compressions, and the number of cases requiring salvage analgesia. Awakening time was defined as the duration from the cessation of anesthetics until the patient responded correctly to external verbal stimuli; extubation time was defined as the duration from the cessation of anesthetics to the removal of the endotracheal tube.

### Statistical analyses

The sample size was determined using PASS 15.0. Based on the pilot experiment results, the mean FLACC or NRS scores at the four time points in the four groups were 1.1, 1.9, 2.7, and 1.8, respectively, with a standard deviation of 1.2. Assuming α = 0.05 and β = 0.2, a sample size of 14 patients per group was calculated. Considering a 20% potential loss to follow-up rate, 18 patients were targeted for enrollment in each group. Utimately, we enrolled 20 patients in each group, resulting in a total of 80 patients.

SPSS 26.0 software was used for statistical analysis. Normally distributed continuous data were expressed as mean ± standard deviation. Non-normally distributed continuous data were expressed as the median and quartile (25th-75th percentile). Categorical data were expressed as frequency (percentage).

Non-normally distributed continuous data like age, height, weight were statistically analyzed using Kruskal-Wallis H test and multiple comparisons were performed using Bonferroni method. Categorical data like ASA grade, type of surgical distribution, choking or agitation during extubation were statistically analyzed using the χ² test or Fisher’s exact probability method, and multiple comparisons were performed using the Z-test with the *P*-value adjusted by the Bonferroni method (the grouping needs to be put into columns and the event into rows when performing the Z-test). The pain or sedation scores at different postoperative time points in this study were non-normally distributed data, so generalized estimating equations were used for statistical analysis. *P<* 0.05 were considered statistically significant.

## Results

### Demographic, surgery, and anesthesia data

**Fig. 1:**
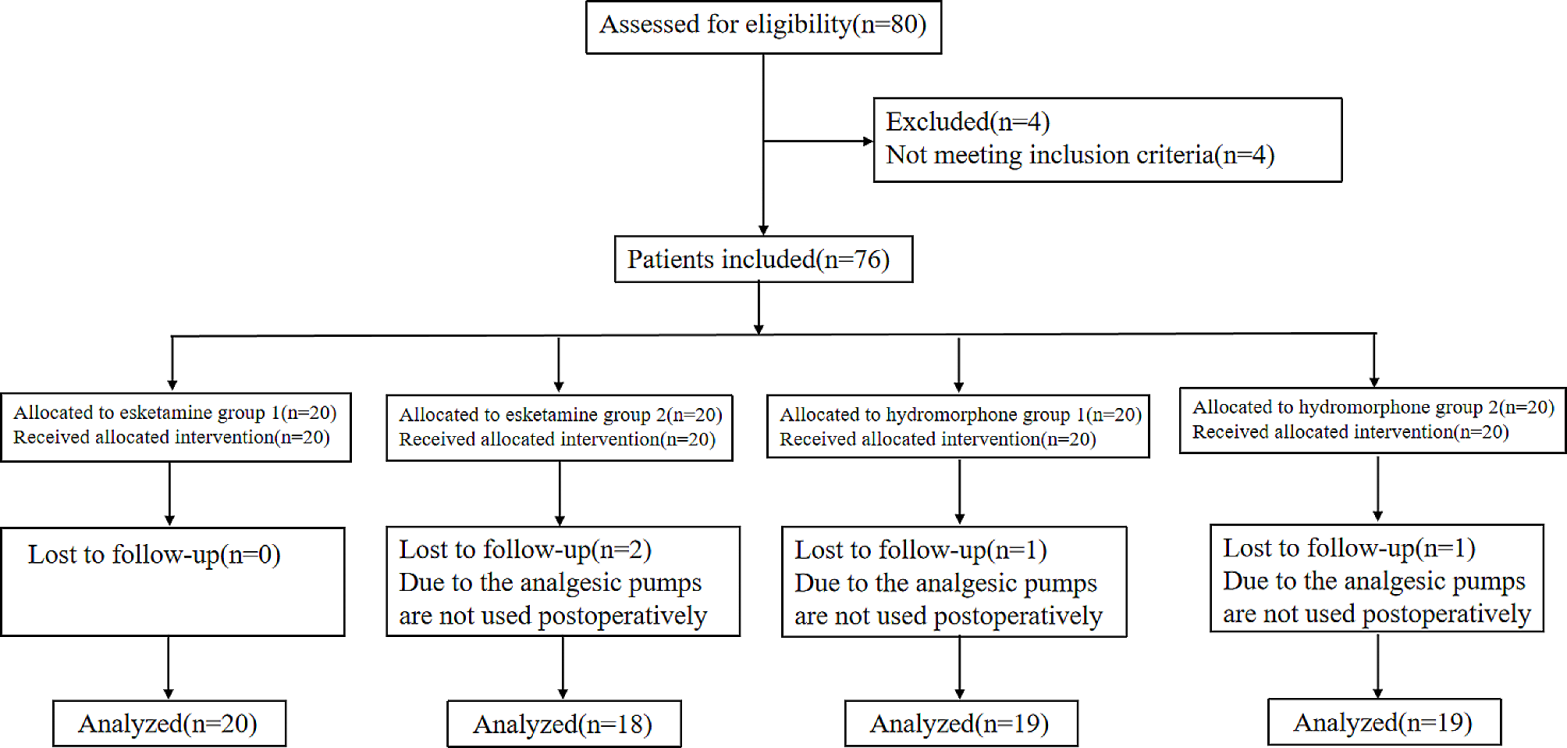
Consolidated standards of reporting trials flow diagram

In this study, a total of 80 male patients were initially included. However, four patients were later excluded from the analysis as they did not use an analgesic pump after surgery. Consequently, the final analysis comprised four groups: esketamine group 1 (ES1) with 20 patients, esketamine group 2 (ES2) with 18 patients, hydromorphone group 1 (H1) with 19 patients, and hydromorphone group 2 (H2) with 19 patients (Fig. 1).

The baseline characteristics including age, height, weight, ASA status, intraoperative remifentanil consumption, blood loss and fluid infusion, as well as type of surgical distribution were comparable between the four groups, as shown in Table 1.

**Table 1:** Comparison between groups of general data and intraoperative conditions

Group	ES1 group(*n* = 20)	ES2 group(*n* = 18)	H1 group(*n* = 19)	H2 group(*n* = 19)	*P* value
Age (yr)	6.5[3.0-8.8]	3.0[2.0–6.0]	3.0[2.0–6.0]	4.0[3.0–8.0]	0.274
Height (cm)	124.0[97.0-139.8]	100.0[91.5-117.5]	102.0[96.0-120.0]	108.0[95.0-135.0]	0.297
Weight (kg)	24.5[15.0-39.8]	19.0[14.3–25.3]	17.0[15.0–25.0]	18.0[16.0–35.0]	0.404
ASA status ( I/II), N	17/3	15/3	15/4	15/4	0.957
Remifentanil consumption (ug)	358.0[191.0-536.8]	346.5[245.8-488.8]	394.0[228.0-500.0]	300.0[234.0-550.0]	0.965
Blood loss (ml)	5.0[4.0–6.0]	5.0[3.8–8.3]	5.0[3.0–6.0]	3.0[3.0–5.0]	0.073
Infusion volume (ml/h)	135.7[99.2-203.4]	107.5[89.9-131.5]	104.3[83.9-134.3]	121.6[68.2-160.7]	0.251
Type of surgery					0.548
Penile or urethroplasty (I/II), N	7/1	6/0	4/1	5/1	
Penile correction (I/II), N	5/0	1/0	4/0	4/0	
Penile or urethroplasty combined with penile straightening (I/II), N	5/2	7/1	6/0	6/1	
Laparoscopic pyeloplasty (I/II), N	0/0	1/2	1/3	0/2	

Data are presented as median (25th-75th percentile) or numbers(n/n). ASA: American society of anesthesiologists.

### Primary outcome

**Fig. 2:**
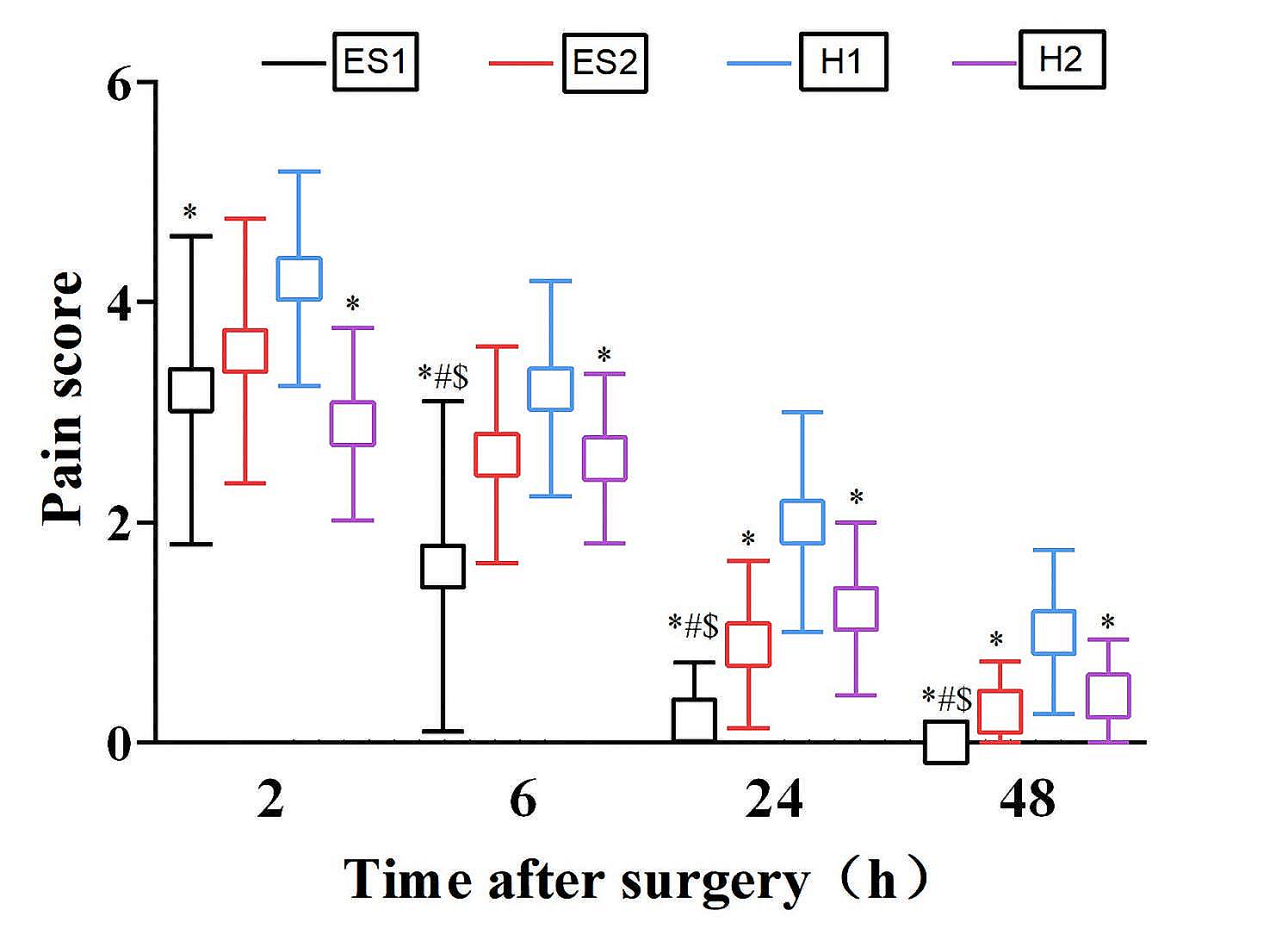
Comparison between groups of pain score at each time point. *P < 0.05 compared with H1 group; ^#^*P* < 0.05 compared with H2 group; ^$^*P* < 0.05 compared with ES2 group

FLACC or NRS scores are commonly used to evaluate postoperative pain levels. In this study, pain assessment was performed at 2, 6, 24, and 48 h postoperatively by choosing the appropriate assessment method according to the patient’s age. Figure 2 presents the comparison results of pain scores at each time point after surgery in ES1 group, ES2 group, H1 group and H2 group.

Compared to the H1 group, the pain scores at each time point after surgery were significantly lower in the ES1 group and the H2 group, with statistically significant difference. The ES2 group had lower pain scores than the H1 group only at 24 and 48 h postoperatively, whereas the difference in pain scores at 2 and 6 h postoperatively was not statistically significant.

When compared with the H2 group, pain scores were significantly lower in the ES1 group at 6, 24, and 48 h postoperatively. However, there was no significant difference in pain scores between the H2 group and ES2 group at each time point after surgery.

Furthermore, when compared with the ES2 group, the pain scores at 6, 24, and 48 h postoperatively were significantly reduced in the ES1 group, with statistically significant differences.

### Secondary outcomes

**Fig. 3:**
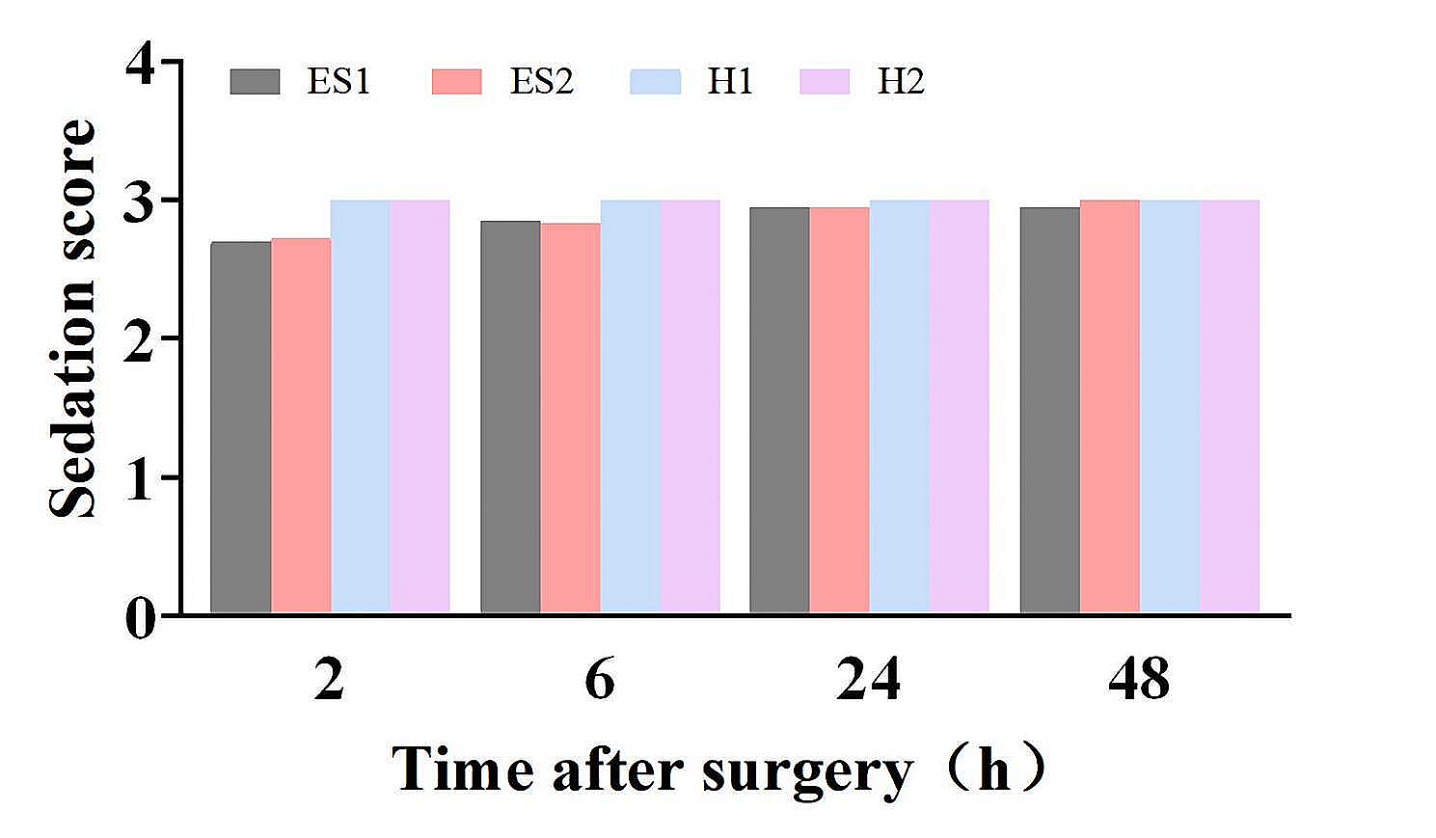
Comparison between groups of sedation score at each time point

Postoperative sedation scores showed no significant differences among the ES1 group, ES2 group, H1 group and H2 group (Fig. 3). Additionally, there were no significant differences observed in extubation time, awakening time, incidence of choking cough at extubation, or the need for postoperative salvage analgesia between the four groups (Table 2).

The incidence of agitation at extubation was 45% (9/20) in the ES1 group, 22% (4/18) in the ES2 group, 11% (2/19) in the H1 group, and 11% (2/19) in the H2 group, and the differences between the groups were statistically significant (*P* = 0.041). However, the results of multiple comparisons showed no statistically significant difference between each two groups (Table 2).

The difference in time to first postoperative PCA was statistically significant among the four groups (*P* = 0.021). The time to first postoperative PCA was longer in the ES1, ES2 and H2 groups than in the H1 group, and multiple comparisons only showed statistically significant differences between the ES2 and H1 groups (Table 2).

Moreover, the difference in the total and effective number of PCA compressions between the four groups was statistically significant (*P* < 0.001). When compared with the H1 group, both the ES1 group and the ES2 group demonstrated a significantly reduced total and effective number of PCA compressions. There were no significant differences in pairwise comparisons between the ES1, ES2, and H2 groups (Table 2).

During the 48-hour postoperative follow-up, adverse reactions were observed in some patients. In the ES1 group, two patients experienced nausea, and one patient reported drowsiness, resulting in an adverse reaction incidence of 15% (3/20). In the ES2 group, three cases of nausea and one case of vomiting were recorded, leading to an adverse reaction incidence of 22% (4/18). The H1 group had seven cases of nausea, three cases of vomiting, and one case of drowsiness, with an adverse reaction incidence of 58% (11/19). In the H2 group, there were six cases of nausea and two cases of vomiting, resulting in an adverse effect incidence of 42% (8/19). In addition, esketamine related neurologic symptoms were not recorded.

A significant difference was observed in the incidence of adverse effects among the groups within 48 h after surgery (*P* = 0.021), with the ES1 group showing a significantly reduced incidence of adverse reactions compared to the H1 groups (Table 3). It is important to note that the above-mentioned adverse symptoms were mild and did not require drug treatment.

**Table 2:** Comparison of anesthetic emergence quality and analgesic efficacy between groups

Group	ES1 group(*n* = 20)	ES2 group(*n* = 18)	H1 group(*n* = 19)	H2 group(*n* = 19)	*P* value
Extubation time(min)	14.5[13.0-20.5]	16.0[11.8–18.5]	17.0[10.0–22.0]	20.0[11.0–26.0]	0.666
Awakening time(time)	11.5[8.0-14.5]	9.5[5.0-12.5]	9.0[5.0–16.0]	8.0[6.0–16.0]	0.629
Choking cough at extubation, n/total N(%)	2/20(10%)	3/18(17%)	2/19(11%)	2/19(11%)	0.897
Agitation at extubation(n), n/total N(%)	9/20(45%)	4/18(22%)	2/19(11%)	2/19(11%)	0.041
Postoperative salvage analgesia, n/total N(%)	5/20(25%)	3/18(17%)	2/19(11%)	3/19(16%)	0.700
The time of first PCA(min)	70.0[60.0-100.0]	75.0[60.0-87.5]^*^	52.5[47.5–60.0]	60.0[50.0–70.0]	0.021
The total number of PCA compressions(times)	3.0[2.0-4.3]^*^	4.0[3.0–4.0]^*^	8.0[5.0–8.0]	5.0[4.0–6.0]	<0.001
The effective number of PCA compressions(times)	3.0[2.0-4.3]^*^	4.0[3.0–4.0]^*^	8.0[5.0–8.0]	5.0[4.0–6.0]	<0.001

Data are presented as median (25th-75th percentile) or numbers(n/total). ^*^P < 0.05 compared with H1 group. PCA: patient-controlled analgesia

**Table 3:** Comparison of the incidence of adverse reactions within 48 h after surgery between groups

Group	Number of examples, n/total N(%)	Nausea(n)	Vomiting(n)	Drowsiness(n)
ES1(*n* = 20)	3/20(15%)^*^	2	0	1
ES2(*n* = 18)	4/18(22%)	3	1	0
H1(*n* = 19)	11/19(58%)	7	3	1
H2(*n* = 19)	8/19(42%)	6	2	0

Data are presented as numbers(n/total). ^*^P < 0.05 compared with H1 group

## Discussion

Our study findings suggest that the combination of low-dose esketamine in analgesia pumps effectively alleviates postoperative pain in pediatric urology patients, leading to a reduced number of analgesic pump compressions and a longer time to first PCA use after surgery. Utilizing esketamine in anesthesia induction combined with analgesia pumps appears to be a favorable administration method as it significantly reduces the incidence of postoperative adverse events.

Intraoperative intravenous administration of 0.3 mg/kg esketamine significantly decreased pain scores and anxiety scores in patients undergoing breast or thyroid surgery [16]. Chen Sai et al. found that exposure to esketamine was one of the independent risk factors for emergence delirium (ED) during recovery from anesthesia in preschool children. To reduce the incidence of ED, the dose of esketamine used during induction of anesthesia should be ≤ 0.3 mg/kg [17]. Therefore, the dose of esketamine at the induction of anesthesia in this study was set at 0.3 mg/kg. Previous studies have found that postoperative continuous infusion of esketamine 0.015 mg/kg/h is comparable to that of 0.125 mg/kg/h, and the incidence of postoperative psychosis is lower [18]. In conjunction with the pre-trial, we compounded a lower dose of esketamine in the analgesic pump for postoperative multimodal analgesia. The background infusion dose range of hydromorphone for postoperative PCA in pediatric patients was 1–3 µg/kg/h, and the button dose range was 1–3 µg/kg (43%) and 4–6 µg/kg (40%) [19]. Therefore, in the present study in groups H1 and H2 hydromorphone background infusion was set at 1 µg/kg/h and 1.5 µg/kg/h, respectively, and analgesic pump button dose was set at 2 µg/kg and 3 µg/kg, respectively.

Introduced in 1970, ketamine is well known for its various pharmacological effects, including anesthetic, analgesic, sedative, anti-inflammatory and antidepressant properties [20]. Esketamine, as the S-enantiomer of ketamine, shares similar pharmacological effects. Our findings align with previous studies, where combining low-dose esketamine intravenous infusion in analgesia pumps was associated with improved postoperative analgesia. Ying Zhang et al. found the application of esketamine-dexmedetomidine in an analgesic pump in patients undergoing scoliosis correction significantly reduced the incidence of moderate to severe pain within 72 h and the incidence of moderate-to-severe postoperative pain was reduced by 24% [21]. Esketamine alone in analgesia pumps has also been shown to provide effective postoperative pain relief [22, 23].

Our study observed a possible opioid-sparing effect of esketamine.The analgesic effect of the ES2 group was comparable to that of the H2 group and superior to that of the H1 group, suggesting that the addition of esketamine to the analgesic pump significantly relieved the patients’ postoperative pain while reducing the use of hydromorphone. In addition, we noted a significant reduction in the incidence of adverse effects in the esketamine group, particularly nausea and vomiting. Similar to the results of the mata analysis, the addition of ketamine to the analgesic pump could link bar postoperative pain, reduce opioid use, and decrease the incidence of postoperative nausea and vomiting [24]. Our previous findings also demonstrated the opioid-reducing effects of esketamine in thoracic surgery patients [25].

Regarding sedation scores, no significant differences were observed at each time point after surgery between the esketamine and hydromorphone groups. Perioperative administration of low-dose esketamine did not lead to significant sedative effects, and no esketamine-associated side effects were observed. Moreover, the incidence of agitation at extubation in the ES1 group was slightly higher than in the other three groups, but pairwise comparisons did not yield statistical significance. Thus, it is not indicative that administering 0.3 mg/kg esketamine at induction of anesthesia increases the incidence of agitation during extubation. Other factors may have influenced these results.

Although previous studies have shown that esketamine can prolong anesthesia recovery time in adults [26], we did not observe any differences in the time to extubation and awakening time among the four groups in children. Ketamine clearance in children was twice as high as in adults, differences in enzyme metabolism between children and adults may explain the disparate effects of esketamine on anesthesia recovery time in the two populations [27].

In our study, we introduced the use of esketamine at a dose of 1.0 mg/kg in conjunction with an analgesic pump. The study revealed promising outcomes when esketamine was incorporated into hydromorphone analgesic pumps, administered at a continuous background rate of 0.01 mg/kg/h, resulting in effective analgesia. Furthermore, this investigation delves into establishing optimal esketamine administration protocols for pediatric patients undergoing urological surgery, contributing to the development of clinical guidelines in this specific medical context. However, this study has certain limitations. The secondary objective was to explore the rational mode of esketamine administration, but the comparison was limited to esketamine in anesthesia-induced combined analgesia pumps versus esketamine pumps alone. Not including a group with esketamine only administration during anesthesia induction makes the conclusions somewhat controversial. Additionally, acute pain carries a risk of transitioning to chronic pain [28]. Ketamine has clear benefits in the prevention and treatment of chronic pain [29], and longer follow-up periods are needed to examine esketamine’s effect on the incidence of chronic pain.

## Conclusions

In conclusion, our study supports the use of low-dose esketamine infusion in analgesia pumps to effectively relieve postoperative pain in pediatric urology patients and reduce the number of analgesic pump compressions. The perioperative administration method of combining anesthesia induction with esketamine infusion in analgesia pumps is recommended. However, further studies with larger sample sizes and longer follow-up periods are necessary to fully assess the impact of esketamine on chronic pain incidence.

## Data Availability

The datasets used and/or analysed during the current study are available from the corresponding author on reasonable request.

## Abbreviations

FLACC: Face, Leg, Activity, Crying, and Comfort
NRS: Numerical Rating Scale
PCA: patient-controlled analgesia
NMDA: N-methyl-D-aspartate
ASA: American Society of Anesthesiologists
HR: heart rate
BP: blood pressure
SPO_2_: pulse oximetry
P_ET_CO_2_: respiratory end-tidal carbon dioxide
ED: emergence delirium

## References

[1] Wilson CA, Sommerfield D, Drake-Brockman TFE, Lagrange C, Ramgolam A, von Ungern-Sternberg BS. A prospective audit of pain profiles following general and urological surgery in children. Paediatr Anaesth. 2017;27(11):1155–64. 10.1111/pan.13256. 10.1111/pan.13256 29030932

[2] Nikolajsen L, Brix LD (2014). Chronic pain after surgery in children. Curr Opin Anaesthesiol.

[3] Fortier MA, Chou J, Maurer EL, Kain ZN (2011). Acute to chronic postoperative pain in children: preliminary findings. J Pediatr Surg.

[4] Morrison K, Herbst K, Corbett S, Herndon CD (2014). Pain management practice patterns for common pediatric urology procedures. Urology.

[5] Plein LM, Rittner HL (2018). Opioids and the immune system - friend or foe. Br J Pharmacol.

[6] Colvin LA, Bull F, Hales TG (2019). Perioperative opioid analgesia-when is enough too much? A review of opioid-induced tolerance and hyperalgesia. Lancet.

[7] Cravero JP, Agarwal R, Berde C, Birmingham P, Coté CJ, Galinkin J, Isaac L, Kost-Byerly S, Krodel D, Maxwell L et al. The Society for Pediatric Anesthesia recommendations for the use of opioids in children during the perioperative period. Paediatr Anaesth 2019, 29(6):547–7110.1111/pan.13639. 10.1111/pan.13639 PMC6851566 30929307

[8] Mayoral Rojals V, Charaja M, De Leon Casasola O, Montero A, Narvaez Tamayo, Varrassi G (2022). New insights into the Pharmacological Management of Postoperative Pain: a narrative review. Cureus.

[9] Chou R, Gordon DB, de Leon-Casasola OA, Rosenberg JM, Bickler S, Brennan T, Carter T, Cassidy CL, Chittenden EH, Degenhardt E (2016). Management of Postoperative Pain: a clinical practice Guideline from the American Pain Society, the American Society of Regional Anesthesia and Pain Medicine, and the American Society of Anesthesiologists’ Committee on Regional Anesthesia, Executive Committee, and Administrative Council. J Pain.

[10] Wang J, Huang J, Yang S, Cui C, Ye L, Wang SY, Yang GP, Pei Q (2019). Pharmacokinetics and safety of Esketamine in Chinese patients undergoing painless gastroscopy in comparison with ketamine: a randomized, open-label clinical study. Drug Des Devel Ther.

[11] Crellin, Harrison, Santamaria N, Babl (2015). Systematic review of the Face, Legs, Activity, Cry and Consolability scale for assessing pain in infants and children: is it reliable, valid, and feasible for use?. Pain.

[12] Tsze DS, von Baeyer CL, Pahalyants V, Dayan PS (2018). Validity and reliability of the Verbal Numerical Rating Scale for Children aged 4 to 17 years with Acute Pain. Ann Emerg Med.

[13] Keles S (2018). Comparison of oral dexmedetomidine and midazolam for premedication and emergence delirium in children after dental procedures under general anesthesia: a retrospective study. Drug Des Devel Ther.

[14] Lozano-Díaz D, Valdivielso Serna A, Garrido Palomo R, Arias-Arias Á, Tárraga López PJ, Martínez Gutiérrez A (2021). Validation of the Ramsay scale for invasive procedures under deep sedation in pediatrics. Paediatr Anaesth.

[15] Pan Y, Wang Y, Lie D, Liu D, Chen X, Wu Z, Chen L, Wang H, Peng L, Liang H (2021). Effectiveness of analgesia with hydromorphone hydrochloride for postoperative pain following surgical repair of structural congenital malformations in children: a randomized controlled trial. BMC Anesthesiol.

[16] Zhou D, Liu F, Jiang F, Ye X, Gong X, Zhang M (2023). Sub-anesthesia Dose of S-Ketamine reduces Postoperative Pain and anxiety in patients receiving breast and thyroid surgery: a Randomized, Controlled Trial. Pain Physician.

[17] Chen S, Yang JJ, Zhang Y, Lei L, Qiu D, Lv HM, Sun ZT, Hashimoto K, Yang JJ (2023). Risk of esketamine anesthesia on the emergence delirium in preschool children after minor surgery: a prospective observational clinical study. Eur Arch Psychiatry Clin Neurosci.

[18] Bornemann-Cimenti H, Wejbora M, Michaeli K, Edler A, Sandner-Kiesling A (2016). The effects of minimal-dose versus low-dose S-ketamine on opioid consumption, hyperalgesia, and postoperative delirium: a triple-blinded, randomized, active- and placebo-controlled clinical trial. Minerva Anestesiol.

[19] Nelson KL, Yaster M, Kost-Byerly S, Monitto CL (2010). A national survey of American Pediatric anesthesiologists: patient-controlled analgesia and other intravenous opioid therapies in pediatric acute pain management. Anesth Analg.

[20] Gao M, Rejaei D, Liu H (2016). Ketamine use in current clinical practice. Acta Pharmacol Sin.

[21] Zhang Y, Cui F, Ma JH, Wang DX (2023). Mini-dose esketamine-dexmedetomidine combination to supplement analgesia for patients after scoliosis correction surgery: a double-blind randomised trial. Br J Anaesth.

[22] Min M, Du C, Chen X, Xin W (2023). Effect of subanesthetic dose of esketamine on postoperative rehabilitation in elderly patients undergoing hip arthroplasty. J Orthop Surg Res.

[23] Xu Y, Chen Q, Li P, Song X (2023). Safety and efficacy of esketamine for postoperative analgesia in pediatric patients with hypospadias. Front Surg.

[24] Wang L, Johnston B, Kaushal A, Cheng D, Zhu F, Martin J (2016). Ketamine added to morphine or hydromorphone patient-controlled analgesia for acute postoperative pain in adults: a systematic review and meta-analysis of randomized trials. Can J Anaesth.

[25] Yuan J, Chen S, Xie Y, Wang Z, Xing F, Mao Y, Wang J, Yang J, Li Y, Fan X (2022). Intraoperative Intravenous Infusion of Esmketamine has opioid-sparing effect and improves the quality of recovery in patients undergoing thoracic surgery: a Randomized, Double-Blind, placebo-controlled clinical trial. Pain Physician.

[26] Zhang C, He J, Shi Q, Bao F, Xu J (2022). Subanaesthetic dose of esketamine during induction delays anaesthesia recovery a randomized, double-blind clinical trial. BMC Anesthesiol.

[27] Zanos P, Moaddel R, Morris PJ, Riggs LM, Highland JN, Georgiou P, Pereira EFR, Albuquerque EX, Thomas CJ, Zarate CA. Jr. : ketamine and ketamine metabolite pharmacology: insights into therapeutic mechanisms. Pharmacol Rev 2018, 70(3):621–6010.1124/pr.117.015198. 10.1124/pr.117.015198 PMC6020109 29945898

[28] Fletcher D, Stamer UM, Pogatzki-Zahn E, Zaslansky R, Tanase NV, Perruchoud C, Kranke P, Komann M, Lehman T, Meissner W (2015). Chronic postsurgical pain in Europe: an observational study. Eur J Anaesthesiol.

[29] Culp C, Kim HK, Abdi S (2020). Ketamine use for Cancer and Chronic Pain Management. Front Pharmacol.

